# Gynecological cancer inpatients need more supportive nursing care than outpatients: a comparative study

**DOI:** 10.1186/s12912-019-0355-x

**Published:** 2019-08-16

**Authors:** Tina Mawardika, Yati Afiyanti, Hayuni Rahmah

**Affiliations:** Department of Maternity Nursing, Faculty of Nursing, Universitas Indonesia, FIK UI Campus, Jl. Prof. Dr. Bahder Djohan, Depok, West Java 16424 Indonesia

**Keywords:** Supportive nursing care needs, Inpatient, Gynecological cancer

## Abstract

**Background:**

Gynecological cancer inpatients and outpatients may have distinct supportive nursing care needs. This study aimed to compare the supportive care needs between these two patient cohorts.

**Methods:**

This cross-sectional comparison analytic study aimed to identify the differences between the supportive nursing care needs of the gynecological cancer inpatients and outpatients. Data were collected from 200 participants who were recruited through consecutive sampling method.

**Results:**

The results showed that gynecological cancer inpatients needed more supportive nursing care than the outpatients. The most reported supportive nursing care needs of the inpatients were in the domains of physical (80%) and the psychological (84%). Whilst, the outpatients needed more health information support (78%). There was a significant difference between the supportive nursing care needs of gynecological cancer inpatients and outpatients (*p* value = 0.001). Supportive nursing care needs of the inpatients were 44 times higher compared to those of the outpatients.

**Conclusions:**

The gynecological cancer inpatients and outpatients need supportive nursing care differently. Therefore, nurses should assess supportive care needs of their patients early during the care in each setting so that the intervention could be tailored to the patient’s individual needs. Our study findings can help nurses navigate the supportive care needs for gynecological cancer patients receiving inpatient and outpatient care.

## Background

The number of gynecological cancer deaths in Indonesia has been increasing, largely as a consequence of the lack of knowledge on the risks of cancer and inadequate access to health care service [1]. The nursing care needs of gynecological cancer patients vary based on the condition and the activities of the patient. Patients receive inpatient care mainly to improve their general health condition and to attend scheduled therapies that require certain time for preparation and follow-up in the medical facility. On the other hand, cancer patients can also have a scheduled therapy program such as oral chemotherapy without hospitalization [1]. The differences between inpatient and outpatient care imply varying nursing care needs of the cancer patients [1]. These two groups of cancer patients may necessitate different supportive care to meet their physical, psychological, social, spiritual, sexual, and practical information needs [2].

Unfulfilled supportive care needs may lead to multidimensional problems that are interrelated and overlapping [3]. Physical issues including fatigue, pain, and nausea are the most commonly reported issues in gynecological cancer patients. Furthermore, gynecological cancer patients often suffer from psychological problems such as depression, anxiety, and nervousness caused by the cancer diagnosis and treatments. Patients diagnosed with cervical cancer commonly experience anxiety and feel that they are going to die in a devastating condition. Such psychological problems may also bring about social problems. Frequent social drawbacks associated with cervical cancer patients include social isolation, role dysfunction, dependency, and loss of productivity. These major problems are some instances to illustrate how unmet supportive care needs are linked to the low quality of life of the cancer patients. Therefore, assessing the supportive nursing care needs of the gynecological cancer inpatients and outpatients is of paramount importance if we are to improve the patients’ quality of life [4]. This study aimed to identify the differences of supportive nursing care needs between gynecological cancer inpatients and outpatients patients at Surakarta Hospital in Solo, Central Java, Indonesia.

## Methods

This study was a cross-sectional comparative study of the gynecological cancer patients receiving inpatient and outpatient care. Participants were recruited by using consecutive sampling method at a hospital in Solo, Central Java, Indonesia, during May 2017. Patients attending the inpatient and outpatient oncology units of this hospital were approached by the first author. A patient was deemed eligible for the study if she met the criteria of: (1) having been diagnosed with gynecological cancer of any type and stage, (2) having completed primary cancer treatment (surgery, chemotherapy, and/or radiation), (3) having no current cancer recurrence in the primary site or other areas, (4) being able to communicate in Bahasa Indonesia, (5) being married, and (6) being able to give consent to participate in the study. A total of 200 participants agreed to take part in this study.

We used the paper-based Supportive Care Needs Survey (SCNS-SF34) to measure the supportive care needs of the patients. This questionnaire consists of 34 question items under 5 domains, i.e.: physical, psychological, care support, health system and information, and sexuality needs. This tool has demonstrated high validity and reliability (validity score = 0.302–0.792; Cronbach alpha = 0.933) [5]. Ethical approvals were issued by the Institutional Review Boards of Faculty of Nursing, Universitas Indonesia and Surakarta Hospital. We adhered to the ethical principles to protect the participant’s right to self-determination, free of discomfort and harm related to the study, as well as the anonymity and confidentiality of the data.

### Data analysis

The socio-demographic, cancer-related characteristics, and SCNS-SF34 data were calculated for the descriptive analysis of this study. A bivariate analysis using chi-square test was conducted to obtain the differences of supportive nursing care needs between gynecological cancer inpatients and outpatients. Lastly, we performed a logistic regression to determine the factors which mostly accounted for the supportive care needs of the patients. All data analysis was conducted using SPSS software (version 17, SPSS Inc., Chicago, IL, USA). *P* value was set at < 0.05 for the statistical significance.

## Results

### Participants characteristic

Table 1 shows that the majority of the respondents in this study were homemakers, had lower educational background, and had family income lower than the minimum average income. Median age of the outpatient patients was 52 years old, while the inpatient patients were aged 54 years.

**Table 1:** The Characteristics of Respondents Based on Education, Income, Occupation, and Age, Surakarta in May 2017 (*n* = 200)

Characteristics	Group			
	Inpatient (n = 100)		Outpatient (*n* = 100)	
	Frequency (n)	Percentage (%)	Frequency (n)	Percentage (%)
Education				
Junior high school	64	(64%)	59	(59%)
Senior High school	36	(36%)	41	(41%)
Income				
>Minimum Wage	35	(35%)	17	(17%)
≤Minimum Wage	65	(65%)	83	(83%)
Occupation				
Working	44	(44%)	28	(28%)
Not working	56	(56%)	72	(72%)
Age (Year)				
x (SD)	52.6 (5.56)		53.9 (4.81)	
Median	52.0		54.0	
Range	26–68		35–65	

As can be seen in Table 2, the OR = 43.9 indicates that the inpatient patients had 43.9 times higher supportive care needs than those having outpatient care.

**Table 2:** The Difference of Proportion in Supportive Nursing Care Needs for Inpatient and outpatient Gynecological Cancer Patients at the Admission and Outpatient Department for Each Domain, Surakarta, May 2017 (*n* = 200)

Supportive Nursing care Requirement	Treatment Status				Total			
	Inpatient		Outpatient		N	%	*P-Value*	OR
	n	%	N	%				CI 95%
Not Required	60	60	17	17	87	46.7	0.001	43.941 (19.044–101.389)
Required	40	40	83	83	123	53.3		
Total	100		100		200	100		

In general, there was a significant difference between the supportive care needs in the physical and psychological domains which were higher among the inpatient patients than in the outpatient patients (Table 3). The supportive care needs in the health information and system domain were not significantly different between the inpatient and outpatient patients (OR = 2.282). The physical and psychological supportive care needs of the inpatient and outpatient patients were significantly different (OR = 36.00 and 19.75, respectively). Whereas, the supportive care needs of the inpatient and outpatient patients in the domains of care support and sexuality had no significant differences (OR = 0.089 and 0.645, respectively).

**Table 3:** The Difference of Proportion in Supportive Nursing Care Needs for Genealogical Cancer Patients at the Admission and Outpatient Department for Each Domain Hospital, Surakarta in May 2017 (*n* = 200)

Variables	Group				Total			
	Outpatient		Inpatient		N	%	*P-Value*	OR
								CI 95%
	N	%	N	%				
Health System and Information Domain								
Not Required	22	22	11	11	33	16.5	0.057	2.282 (1.041–5.003)
Required	78	78	89	89	167	83.5		
Physical Domain								
Not Required	90	90	20	20	110	55.0	0.000	36.000 (15.909–81.465)
Required	10	10	80	80	90	45.0		
Nursing care Support Domain								
Not Required	59	59	46	46	105	52.5	0.089	1.689 (0.965–2.957)
Required	41	41	54	54	96	47.5		
Psychological Domain								
Not Required	79	79	16	16	95	47.5	0.000	19.75 (9.620–40.546)
Required	21	21	84	84	105	52.5		
Sexuality Domain								
Not Required	91	49.2	94	50,8	185	92.5	0.591	0.645 (0.221–1.886)
Required	9	60.0	6	40,0	15	7.5		

## Discussion

The results of the study show that, in general, most of the gynecological cancer inpatients (83%) required supportive nursing care, in contrast with 40% of the outpatients who did not require akin care. The rest of the participants reported their supportive care needs had already been fulfilled, hence no more supportive nursing care demands. These results demonstrate an obvious difference of the supportive nursing care needs between gynecological cancer inpatients and outpatients.

Another key difference was the type of the supportive care needs. Physical and psychological care supports were mostly demanded by the inpatients, while the information support was the predominant need of the outpatients. Our findings are in line with the previous study results by Hubbard, Vening, Walker, Scanlon, and Kyle [5], Bonaichi et al. [2], and Effendy et al. [6] that the inpatients with cancer reported more physical and psychological needs than the outpatients. Similarly, Hubbart et al. also found that the majority of the outpatients (81%) in their study reported unfulfilled information needs [5].

The physical domain in supportive nursing care needs is related to pain, lack of energy/fatigue, and constant discomfort while doing household chores, and the inability to perform an activity. A study by Meir et al. [7] indicated that 30% of cancer patients reported pain when they were diagnosed with cancer and 65–85% of the patients experienced pain as the cancer developed. It is estimated that a third of cancer patients receiving therapy and three-quarters of all cancer patients suffer from pain. The pain experience is varied and unique to the individual. In a prior survey, 81% of cancer patients complained about having two or more pain types while 34% of them reported having more than three pain types. Pain tends to occur more intensely as the illness progresses depending on its primary location. Other factors that contribute to pain are the stage of illness, the presence of metastasis, infection of tumor in the nervous system, the release of chemical mediators by tumor cells, and factors deriving from the patients such as anxiety and depression.

The psychological domain in the supportive nursing care needs include anxiety, depression, distress, sadness, fear of relapse, concern about medical treatment outcome, uncertainty about the future, situation management, perception on a positive outlook, thoughts on death, and concerns or apprehension that may be experienced by the family members. Studies have shown that the psychological complaints of the cancer patients are related to their physical complaints such as pain. The patient’s fear of cancer was found to be related to the pain caused by cancer metastasis. A former study found 69% of their surveyed cancer patients reported severe pain which led them to consider suicide, and 57% of the patients predicted that their life would end in considerable pain [7].

Schmid-Buchi’s study results suggested that the psychological needs of the hospitalized cancer patients were still mostly unfulfilled through supportive nursing care [8]. Consistent with our study results, Schmid-Buchi also found that psychological needs are among the most required needs for the cancer inpatients [8]. Their results also showed that other unfulfilled psychological needs are fear of metastatic cancer (71.8%) and uncertainty about the future (68%) [8]. The present study also found that the problems of fear of metastasis and future uncertainty in the Indonesian gynecological cancer inpatients were of high levels (81 and 82%, respectively). Our study and Schmid-Buchi’s study are comparable since our study participants were being in the late stage of cancer.

Patients with the advanced stage of cancer are more likely to have mental problems. Treatment-related symptoms such as hair loss can be devastating for patients, causing them to feel depressed and anxious that can disrupt the patients’ daily routines. Patients with advanced staged cancer are in a substantial need of having psycho-emotional support from their family members [9]. Cancer progression comes with a growing psychological pressure. As the psychological pressure elevates, the need for emotional and social support from the patient’s closest circle also increases. Late-stage cancer patients necessitate more psycho-emotional patronage from their family to facilitate with a peaceful end-of-life [10].

Increasing unmet supportive nursing care needs of the gynecological cancer inpatients and outpatients can reveal problems in various domains, reflecting the multidimensional impacts of cancer. A cross-sectional study identified that the high level of unmet needs was related to 76.4% of anxiety level increase in cancer patients, hence the importance of addressing the supportive nursing care. Unfulfilled needs were also found to be related to the lack of social support, low income, age, education, marital status, and lack of information about the cancer prognosis [11, 12]. Some clinical factors including the illness severity, metastasis, recurrence, medical treatment, as well as psychological and physical symptoms are also linked with the level of unmet supportive care needs [11, 12].

The gynecological cancer patients receiving outpatient care, on the other hand, mostly needed informational support regarding the health care service. The health system information domain in SCNS-SF34 is about being given written information regarding important aspects of care, information on ways to manage the illness and its side-effects at home, explanation of the medical tests and their results, as well as access to counseling. Hubard et al. in their study on the nursing care needs of the cancer outpatients reported similar results [5]. As many as 81.2% of late-stage cancer patients had unmet health system information needs, particularly with regards to the diagnostic procedures (72%) and side effects of the therapy (69%) [5]. Such condition may be resulted from the limited number of the healthcare professionals and supporting resources.

A study by Tariman, Doorenbos, Schepp, Singhal, and Berry [13] found three priorities of the cancer patient information needs, i.e.: information on cancer prognosis, illness, and treatment. Cancer is chiefly perceived and often manifests itself as a deadly illness so that the cancer patients are mostly concern about their prognosis. Cancer patients need to deal with their fear of the worst scenario before taking up information on the illness and treatments. Cancer patients of particular diagnostic and stage groups tend to have more concern and unmet needs than other groups of cancer patients. Educating patients about their illness, prognosis, and treatments is the key to active patient engagement in cancer care.

Sufficient information on cancer is necessary since the present society is getting more critical toward the healthcare services. With regard to obtaining correct information, the society has changed from being passive to assertive. As a consequence, changes in behavior have become the focus for improvement in healthcare. The availability of accountable and comprehensible information on cancer may help people to have more positive attitude and active participation in cancer control and care. Many patients nowadays may have extensive knowledge on cancer treatment that can nourish their hope to live through cancer and access the suitable health care service to manage with their cancer [14].

The results of this research show no difference in the sexuality domain of supportive nursing care needs. Both gynecological cancer inpatients and outpatients reported the low level of nursing care needs in sexuality. The same results were obtained by Hubbard, Vening, Walker, Scanlon, and Kyle [5] that only few cancer patients demanded supportive nursing care in the sexuality domain (only 12% of the inpatients and 8% of the cancer outpatients). Prior authors who found similar results argued that most Indonesian patients tended to see sexual issue as very personal and sensitive and they did not expect to receive supportive nursing care for such issue.

Gynecological cancer and its treatments can cause changes in physical, psychological, and social dimensions including sexual dysfunction [4, 15]. Results from a study conducted by Huffman, Hartenbach, Carter, Rash, and Kushner [16] revealed that the gynecological cancer patients needed additional information regarding the cancer treatment side effects on their sexual condition. Thus, the health care professionals should give a special attention to address the sexual problems of the gynecological cancer patients [17].

A framework for nursing care needs for cancer patients, according to Fitch [18], consists of three constructs i.e.: human needs, cognitive evaluation, and coping and adaptation. The constructs are the fundamental concepts of how individuals cope with cancer [18]. The human needs construct is based on Maslow’s Hierarchy of Needs Theory, according to which individuals take action daily to ensure every aspect of their needs is fulfilled [18]. The needs and the ways to fulfill them are unique to individuals. Similarly, the supportive nursing care needs of the inpatients and outpatients are also individually defined despite some commonalities. Furthermore,the cognitive evaluation construct is the process whereby an individual perceives and categorizes an event and various aspects of his or her life according to the impacts of the events on his or her welfare [18]. Every individual has a unique response to a situation based on their cognitive evaluation. A coping and adapting construct is an action and a process to manage a pressing event or situation [18].

Our results indicate that there is a statistically significant difference between the supportive nursing care needs of the gynecological cancer inpatients and outpatients (*p* value = 0.001). The needs for supportive nursing care were 43.9 times greater for the hospitalized cancer patients than the outpatients. Our finding is supported by a prior study result in conducted Ireland by Armes et al. [14] which found that inpatients had more supportive nursing care needs than the outpatients. Patients receiving inpatient and outpatient care service are primarily being in different cancer trajectories with their own clinical conditions that entail different supportive care needs. Treatment activities at the medical facilities also affect the supportive care needs of the patients [1].

Supportive nursing care needs refer to all aspects required during treatment with the purpose of improving the life quality of the patients with a life-threatening illness [19]. Gynecological cancer patients have different types of nursing care needs types pertaining to the stage and the nursing care process [20]. Cancer patients undergoing treatment experience many physical, emotional, and social changes such as nausea, fatigue, pain, poor sleep quality, altered self-esteem, anxiety about the treatment outcome, and altered daily routines. Such changes may bring different supportive nursing care needs of the patients. Poor management of the treatment side effects and the inability to perform self-care can affect the life quality of the cancer patients [21–23].

### Nursing implications

Our study findings can help nurses navigate the supportive care needs for gynecological cancer patients receiving inpatient and outpatient care. Patients should be assessed individually for their supportive care needs so that the intervention could be tailored to the patient’s individual needs. Inpatients need more supportive care to address their physical and psychological problems, especially pain, fatigue, and fear of cancer recurrence. On the other hand, to particularly support the patients receiving outpatient care, sources of comprehensive information regarding gynecological cancer should be made accessible for patients and caregivers. Patient’s social circle i.e. family and friends should be engaged to support cancer care of the patients. Nurses can also facilitate the groups of cancer patients to provide peer support for each other not only in terms of information but also emotional and belonging support. Further studies can be aimed for qualitative exploration of the supportive care needs of the gynecological cancer inpatients and outpatients to deepen our understanding on the unveiled aspects of the supportive cancer care needs of these patient cohorts.

## Conclusions and recommendations

There are significant differences between the supportive nursing care needs of the gynecological cancer inpatients and outpatients. The inpatients need more supportive nursing care for their physical and psychological problems than the outpatients. Whilst, outpatients demand more informational support for their cancer care. Patients having inpatient care are usually having more complicated clinical condition or being in the crucial cancer trajectory thus requiring different supportive care needs than their outpatient counterparts.

## Data Availability

Not applicable.
